# Fresh frozen cortical strut allograft in two-level anterior cervical corpectomy and fusion

**DOI:** 10.1371/journal.pone.0183112

**Published:** 2017-08-25

**Authors:** Kuang-Ting Yeh, Ru-Ping Lee, Ing-Ho Chen, Tzai-Chiu Yu, Kuan-Lin Liu, Cheng-Huan Peng, Jen-Hung Wang, Pau-Yuan Chang, Wen-Tien Wu

**Affiliations:** 1 Department of Orthopedics, Hualien Tzu Chi Hospital, Buddhist Tzu Chi Medical Foundation, Hualien, Taiwan; 2 School of Medicine, Tzu Chi University, Hualien, Taiwan; 3 Institute of Medical Sciences, Tzu Chi University, Hualien, Taiwan; 4 Department of Medical Research, Hualien Tzu Chi Hospital, Buddhist Tzu Chi Medical Foundation, Hualien, Taiwan; 5 Department of Radiology, Hualien Tzu Chi Hospital, Buddhist Tzu Chi Medical Foundation, Hualien, Taiwan; George Washington University, UNITED STATES

## Abstract

Anterior cervical corpectomy and fusion (ACCF) is one of the main surgical strategies for the management of multilevel cervical spondylotic myelopathy (MCSM). High complication rates of graft bone fracture, resorption, displacement, and fusion collapse or pseudarthrosis have been previously reported. The strategies to prevent the aforementioned complications include using fresh frozen cortical strut allograft (FFCSA) to keep most of the original bone quality and using additional anterior plate fixation to improve the fusion stability and union rate. In this study, we evaluated 4-year follow-ups for surgical outcomes and analyzed the risk factors of MCSM patients who received 2-level ACCF with FFCSA and titanium dynamic plate fixation. We retrospectively collected preoperative and postoperative radiographic and clinical data of patients from 2005 to 2009; the inclusion criteria were having been diagnosed as MCSM and having received 2-level ACCF with an FFCSA fibular shaft and an anterior dynamic plate. The cervical curvature lordosis improved and the neurogenic function recovered well postoperatively. Visual analog scale for neck pain and neck disability index scores both decreased after 12 and 48 months following surgery. The Japanese Orthopaedic Association score recovery rate at postoperative 4 years was 87.5%. Fusion rates achieved 100% at 12 months. The preoperative Nurick score seemed to be the only significant risk factor correlated with the functional recovery rate at 4 years after the surgery. In conclusion, based on a minimum 4-year follow-up of 2-level ACCF with FFCSA and dynamic titanium plates for patients with MCSM, the surgical results were satisfying and the complication rates were low.

## Introduction

Multilevel cervical spondylotic myelopathy (MCSM) is a common cause of neurologic deficits that decrease quality of life. The surgical strategies of decompression for MCSM include both anterior and posterior approaches [1, 2]. The choice between anterior, posterior, and combined approaches is made on the basis of (1) disc or behind-vertebral-body levels of spinal cord compression, (2) sagittal alignment of cervical spine curvature, (3) location of compressive abnormality, (4) presence of preoperative neck pain, and (5) previous operations [3]. Anterior cervical corpectomy and fusion (ACCF) is advocated as one of the main operation methods for MCSM. In the 1990s, favorable results of long level ACCF reconstructed with cortical strut allograft (CSA) were reported [4], but high complication rates of graft bone fracture, resorption, displacement, and fusion collapse or pseudarthrosis were also reported [5]. The advantage of ACCF is that it can establish direct decompression of myelopathy levels by resecting the ventral constriction and cervical stability, which is conducive to relieving pressure on the compressed cervical cord [6, 7]. The occurrence of the complications of ACCF often result from managements of CSA that decrease the strength of the structure by freeze drying or radiation [8, 9], unstable surfaces between host vertebral bone and CSA, and poor bone quality of host vertebrae [10]. Strategies to prevent the aforementioned complications include using fresh frozen CSA (FFCSA) to keep most of the original bone quality and using additional anterior plate fixation to improve the fusion stability and union rate [11–13]. Additionally, dynamic cervical plate designs are also proven to provide fewer implant complications and faster speed of fusion compared with rigid plate designs [14]. Therefore, ACCF with additional dynamic plate fixation are commonly performed in recent studies [15, 16].

The reconstruction of long-level ACCF needs suitable bone graft carried spacers for corpectomied spaces. The materials used can be divided into metal interbody cages, autogenous strut bone grafts, and allogenous strut bone grafts. ACCF with autogenous strut bone graft has satisfying surgical outcomes but also has a high overall rate of donor site morbidity [17]. The advantages of titanium mesh cages include avoidance of bone grafting procedures and immediate strong anterior column support. Major complications are failure of the cage and plate construct with the need for supplemental posterior stabilization for cases with spasticity or greater than 2-level corpectomies with profound osteoporosis [18]. Allogenous tricorticate iliac crests and fibular shafts for ACCF can act as structural supports to prevent those complications and have better biocompatibility than do metal interbody cages. However, FFCSAs are difficult to achieve and additional operation time is also needed for preparing the FF bones. Consequently, most studies put an emphasis on the surgical results of ACCF with dynamic plates and titanium mesh cages [7, 19]. Earlier studies have demonstrated that CSA for ACCF are mostly performed with rigid plate fixation, which is believed to have higher complication rates [5]. In this study, we explored 4 years of follow-up data on surgical outcomes to analyze the risk factors of MCSM patients who received ACCF with FFCSA and titanium dynamic plate fixation and compared the results with the existing literature.

## Material and methods

### Study design and sampling

This study was approved by the Institutional Review Broad of Hualien Tzu Chi Hospital, Buddhist Tzu Chi Medical Foundation (IRB101-100). We retrospectively collected patient data between January 2005 and December 2009. The inclusion criteria for the patients were a MCSM diagnosis and having undergone a 2-level ACCF in our department. The exclusion criteria were 1) undergoing other spinal surgery methods at the same time; 2) 3 or fewer intervertebral levels of stenosis; 3) engaging in postoperative follow-up less than 48 months; 4) presenting with a history of trauma, neoplasm, infection, or congenital deformations; 5) having had previous surgery of the cervical spine; 6) experiencing a serious chronic systemic illness, such as rheumatoid arthritis or neurodegenerative disease. All patients were clinically and radiographically evaluated before surgery. Clinical evaluation consisted of medical history and physical examination. The clinical results were assessed using the Japanese Orthopaedic Association (JOA) scoring system [20], Nurick score [20], visual analog scale (VAS) for neck pain, and neck disability index (NDI) [21]. At the last follow-up, clinical examination was assessed by the JOA scoring system. The JOA recovery rate proposed by Hirabayashi was also used: recovery rate (RR) = (postoperative JOA score − preoperative JOA score) / (17 − preoperative JOA score) × 100%. The neurological status of the patients at the final follow-up was used to determine clinical prognosis.

Standard anterior-posterior lateral X-rays and magnetic resonance imaging (MRI) of the cervical spine were conducted as preoperative and postoperative radiological evaluation. Cervical curvatures were measured from the inferior C2 endplate to the superior C7 endplate on the lateral X-ray and the positive values were defined as lordosis. MRI was used to evaluate the level of spinal cord compression, which indicates the need for further surgeries and postoperative follow-up of cord expansion condition.

### Preparation of allograft

FFCSA that were used for ACCF were stored at −70°C. Each was of middle fibular diaphysis segments. Infection was ruled out by obtaining specimens for aerobic and anaerobic cultures, and the allografts were placed in a normal saline solution with gentamicin 80 mg/L. The length of the cortical strut bone was measured before surgery. The allografts were prepared to be greater in length than the preoperative measurement of the defect to allow for any intraoperative variations.

### Operative methods

The operation was performed via a left-side anterior approach. The appropriate surgical level was confirmed by intraoperative fluoroscopy. After necessary corpectomies and adjacent discectomies, the posterior longitudinal ligament (PLL) was separated from dura mater using a microdissector. After it had been meticulously separated, it was then removed using a 1.5 mm Kerrison rongeur and microcurettes. After decompression, an adequate-length FFCSA prepared and filled with autogenous bone fragments was impacted into the interbody space and an anterior cervical plate (Zephir) was used to keep the stability of the involved segments. After operation, a hard neck collar (VISTA) was applied for three months with adequate flexion–extension exercise under collar protection.

### Fusion rates and pseudarthrosis

Fusion rates were judged by the absence of motion between the spinous processes on flexion–extension lateral radiographs, the absence of a radiolucent gap between the graft and the endplate, and the presence of continuous bridging bony trabeculae at the graft–endplate junction. All of these criteria must have been met for the patient to be judged solidly fused. A pseudarthrosis was determined radiographically by the absence of bridging osseous trabecular bone from the vertebral bodies to the graft, motion on dynamic radiographs, or the presence of a lucent line at the graft–vertebral body junction. The presence of a pseudarthrosis was evaluated after either a minimum of 1 year of follow-up with the appropriate radiographic signs, or if the patient underwent revision surgery. If any signs of a pseudarthrosis were present, the patients were judged to have a nonunion.

### Statistical analysis

The SPSS software package, version 13.0, was used for statistical analysis. To assess statistical significance, an unpaired Student’s t-test was also performed. The level of statistical significance was set as P < 0.05 was used for comparison of preoperative and postoperative data. Age, gender, preoperative data, smoking history, and diabetes mellitus (DM) status were set as independent variables of JOA recovery rate at postoperative 48 months. To determine the independent variable with the greatest contribution, the stepwise methods of a generalized linear model were applied. The final selection of the independent variables was determined according to the adjusted R-squared value. A statistical analysis was then done on each standard regression coefficient of these independent variables. Those with significance of P < 0.05 were finally selected as the factors influencing the surgical results. The value of the standard regression coefficient for each factor was considered as the magnitude of the impact.

## Results

The included patients were all from Hualien Tzu-Chi Hospital and the surgical procedures were performed by two surgeons, Dr. Wu and Dr. Yu. The fusion and functional results were collected by Dr. Yeh and the success of the fusion was confirmed by a senior radiologist, Dr. Chang.

### Demographic data

A total of 21 men and 29 women were included in this study and their demographic data are presented in Table 1. The mean age of the patients was 58.1 ± 10.1 years old. 13 patients had type 2 DM and 15 of them had recent smoking habits. About half of the patients had a positive Spurling’s sign. 28 patients received C3–6 ACCF (C4 and C5 corpectomy) and 22 patients received C4–7 ACCF (C5 and C6 corpectomy).

**Table 1:** Demographics and preoperative data (N = 50).

	Mean ± SD
Age	58.1 ± 10.1
**Gender**	
Male (%)	21 (42.0)
Female (%)	29 (58.0)
Diabetes mellitus (%)	13 (26.0)
Smoke (%)	15 (30.0)
Spurling sign (%)	26 (52.0)
Operation time (mins)	210.1 ± 38.1
Blood loss (c.c.)	650.0 ± 132.5
Length of stay (days)	9.2 ± 5.7
**Fusion Levels**	
C3-6	28 (56)
C4-7	22 (44)
**Preoperative radiographic data**	
Curvature	7.3 ± 7.2
Segmental angle	3.6 ± 8.0
**Preoperative functional score**	
NDI	33.7 ± 1.4
Nurick score	2.4 ± 0.6
VAS	6.0 ± 0.6
JOA Score	11.2 ± 1.1

Data are presented as n or mean ± standard deviation. NDI = neck disability index; VAS = visual analogue scale; JOA = Japanese orthopedic association.

### Surgical outcomes

The mean operation time was 210.1 ± 38.1 minutes and the average blood loss was 650.0 ± 132.5 cc. All patients were discharged from our hospital smoothly and the mean length of stay was 9.2 ± 5.7 days.

The cervical curvature changed from 7.3 ± 7.2° lordosis to 15.3 ± 7.2° lordosis and the fused segmental angle restored from 3.6 ± 8.0° lordosis to 12.8 ± 6.5° lordosis at postoperative 48 months (Table 2). The mean Nurick score decreased from 2.4 ± 0.6 to 0.3 ± 0.6 and JOA scores improved from 11.2 ± 1.1 to 16.3 ± 0.9 after 48 months. Neck pain VAS changed from 6.0 ± 0.6 to 1.6 ± 0.9 at postoperative 48 months. There were no significant changes of the clinical and radiographical results between postoperative 12 months to 48 months except for NDI: NDI improved from 33.7 ± 1.4 to 8.3 ± 3.1 at postoperative 24 months and maintained to postoperative 48 months. JOA recovery rate at postoperative 4 years was 87.5%. Fusion rates achieved 100% at postoperative 12 months.

**Table 2:** Preoperative and postoperative function status (N = 50).

Item	Preoperative	Postoperative					P-value
		3M	12M	24M	36M	48M	
Curvature	7.3 ± 7.2	15.9 ± 8.9	15.5 ± 8.3	15.3 ± 8.4	15.3 ± 7.3	15.3 ± 7.2	<0.001*
Segmental angle	3.6 ± 8.0	13.1 ± 6.8	12.9 ± 6.6	12.8 ± 6.7	12.8 ± 6.5	12.8 ± 6.5	<0.001*
NDI	33.7 ± 1.4	23.6 ± 1.4	14.4 ± 2.3	8.3 ± 3.1	8.2 ± 3.5	8.2 ± 3.3	<0.001*
Nurick score	2.4 ± 0.6	0.7 ± 0.8	0.3 ± 0.7	0.3 ± 0.7	0.3 ± 0.6	0.3 ± 0.6	<0.001*
VAS	6.0 ± 0.6	2.4 ± 0.7	1.9 ± 0.7	1.9 ± 0.8	1.8 ± 0.8	1.6 ± 0.9	<0.001*
JOA score	11.2 ± 1.1	16.2 ± 1.4	16.3 ± 1.1	16.3 ± 0.8	16.3 ± 0.9	16.3 ± 0.9	<0.001*

M = months; NDI: neck disability index; VAS = visual analogue scale; JOA = Japanese orthopedic association. Data are presented as mean ± standard deviation. P-value: Postoperative 48M vs Preoperative

*P-value<0.05 was considered statistically significant after test

### Predictors of functional recovery rate

JOA recovery rate was set as a dependent variable. Age, DM status, smoking history, preoperative functional scores, existence of preoperative Spurling’s sign, and preoperative radiographic parameters were set as independent variables (Table 3). Preoperative Nurick score seemed to be the only significant risk factor correlated with the JOA recovery rate at 4 years after the surgery (P-value = 0.002). Preoperative absence of Spurling’s sign had a positive impact on the JOA recovery rate with marginal significance (P-value = 0.062).

**Table 3:** Factors associated with the JOA recovery rate at postoperative 48 months. (N = 50).

	Regression Coefficient	95% CI	P-value
Intercept	0.630	(-0.117, 1.377)	0.096
Age	0.002	(-0.001, 0.004)	0.182
**Gender**	-	-	-
Male	0.023	(-0.045, 0.090)	0.501
Female	References	References	NA
**DM**	-	-	-
No	0.022	(-0.039, 0.082)	0.476
Yes	References	References	NA
**Smoke**	-	-	-
No	-0.009	(-0.080, 0.062)	0.803
Yes	References	References	NA
**Spurling sign**	-	-	-
No	0.047	(-0.003, 0.097)	0.062
Yes	References	References	NA
Preoperative Curvature	0.002	(-0.004, 0.007)	0.505
Preoperative Segmental Angle	-0.003	(-0.008, 0.003)	0.332
Preoperative NDI	-0.002	(-0.022, 0.019)	0.868
Preoperative Nurick score	-0.064	(-0.103, -0.025)	0.002*
Preoperative VAS	0.031	(-0.013, 0.075)	0.161

NDI: neck disability index; VAS = visual analogue scale; JOA = Japanese orthopedic association. Dependent variable: JOA recovery rate

*P-value < 0.05 was considered statistically significant after test.

### Complications after operation

Overall incidence of complication rates were 2% (cerebrospinal fluid leakage), 8% (hoarseness), 10% (dysphagia), and 6% (bone graft subsidence). There were no cases of pseudarthrosis. Most complications improved by conservative treatments. The case where cerebrospinal fluid leakage occurred following the operation was treated with lumbar drain and local drain for monitoring. The patient recovered well after one week of observation. Hoarseness and dysphagia mainly occurred in the first two weeks after operation and gradually lessened during the follow-up period. None of the patients needed revision surgeries at 48 months after surgery.

The comparisons between this study and existing ACCF literature with iliac autograft, fibular allograft, and titanium mesh cage are presented in Table 4. The surgical time, estimated blood loss, length of hospital stay, restoration of cervical lordosis, postoperative complication rates, and osseous union rates of the patients in our study were all comparable with previous studies.

**Table 4:** Comparisons of outcomes and fusion rates in their current study with historic studies of ACCF.

	Macdonald et al. [4]	Hirai et al. [6]	Chen et al. [7]	Schnee et al. [17]	Recent study
Fusion material	FSA	ICA or FSA	TMC	ICA	FSA
N	36	39	42	21	50
mean age(y/o)	58.0 ±10.0	59.2 ± 10.7	57.4 ± 7.5		58.1 ± 10.1
OPT(min)	474.0 ± 150.0	211.0 ± 55.3	-	240	210.1 ± 38.1
EBL(ml)	660.0 ± 610.0	340.0 ± 287.0	-	543	650.0 ± 132.5
LOH(day)	27.0 ± 22.0	-	-	8	9.2 ± 5.7
Preop CLA (°)	-	12.8 ± 9.6	16.2 ± 7.0		7.3 ± 7.2
Postop CLA (°)	-	17.7 ± 8.6	22.6 ± 7.5		15.3 ± 7.2
Osseous union	25 (96.2%)	38 (97.4%)	40 (95.2%)	18 (94.5%)	50 (100%)
**Complication**					
Pseudarthrosis	0	1 (0.9%)	2 (4.8%)		0
Kyphotic change	2 (5.6%)	-	2 (4.8%)		0
CSF leakage	3 (8.3%)	-	3 (7.1%)		1 (2%)
Hoarseness	-	0	2 (4.8%)		4 (8%)
Dysphagia	2 (5.6%)	3 (2.6%)	2 (4.8%)		5 (10%)
Bone graft displacement or subsidence	2 (5.6%)	-	-		3 (6%)
Revision surgery	0	0	1 (2.4%)		0

Data are presented as n or mean ± standard deviation. The symbol “-”represented no information. FSA: fibular strut allograft; TMC: titanium mesh cage; ICA: iliac crest autografts; OPT: operation time; EBL: estimated blood loss; LOH: length of hospital stay; CLA: cervical lordotic angle.

## Discussion

This study presents positive surgical outcomes of 2-level ACCF for the patients with MCSM. A JOA recovery rate at 87.5% is comparable to, and the fusion rate of 100% at postoperative 12 months is better than, reports of ACCF in the literature. These favorable outcomes are considered to result from the dynamic titanium anterior plate, the good strength and incorporation of FFCSA, and the preservation of the integrity of upper and lower end plates. Most recent studies have reported that dynamic plate designs provide a faster fusion of the cervical spine in comparison to rigid plate designs [14, 22–24]. FFCSA is known to be a reconstructive biologic option for the spaces of osseous defects that are durable for many decades. The advantages of this reconstruction are that host soft tissue could be attached to the allograft, which could be progressively incorporated by the host [25]. Significantly improved functional outcomes were achieved at postoperative 12 months and maintained to postoperative 48 months in this study. Adequate decompression of the spinal cord and immediate stability of the fused segment were the two key factors of ACCF for the patients with MCSM. The difficulty for ACCF not only lies in the decompression process from ossification of the dura or massive bleeding from the epidural space but also in the reconstruction method after multilevel corpectomies; such reconstruction requires high surgical skill and adequate interbody spacers for fusion. Allogenous cortical strut for ACCF with anterior plate fixation can achieve early stability and fusion. The other important factor for good surgical outcome is the way in which CSA is placed into the corpectomied location. In these cases, we preserved the upper and lower end plates, which are harder surfaces for CSA incorporation and can impart greater stability. The results of favorable fusion rate and with low incidence rate of pseudarthrosis are preferable to those of a previous study involving notched fibular bone graft locked into the recesses of vertebral bodies [26].

The surgical trauma of ACCF and incidence of complications, such as epidural hematoma, cerebrospinal fluid leakage, and bone graft early displacement or subsidence, significantly increase with the number of involved segments [16]. High graft-related complication rates of bone graft fracture, graft pistoning, graft dislodgement, hardware failure, and pseudarthrosis were reportedly related to higher level corpectomies [27]. Higher fusion rates are key factors for decreasing postoperative neck pain arising from pseudarthrosis or kyphotic collapse and maintaining the results of functional recovery. We used FFCSA for filling the corpectomied space and fixed the structure with an anterior dynamic plate so that the fusion rate achieved 100% at 12 months following surgery.

The main complications in this study included cerebrospinal fluid leakage, hoarseness, dysphagia, and bone graft subsidence. The overall complication rate in this study was not higher than previous literature reports. Transient hoarseness and dysphagia after ACCF mainly occurred in the early postoperative period and spontaneously recovered. The observed complications are often caused by endotracheal intubation, laryngeal edema or spasm, symptomatic hematoma, or laryngeal vagus nerve injury. Subsidence of the bone graft occurred in 3 patients but they all achieved good fusion without further collapse and had favorable neurogenic recovery without deterioration at the 4-year follow-up. Dynamic cervical plates, which provide better load sharing while providing overall resistance to motion, overcome the biomechanical limitations of rigid plates [28]. Mild subsidence can be considered as a process of bone incorporation between FFCSA and the end plates.

In conclusion, after a minimum 4-year follow-up of 2-level ACCF with FFCSA and dynamic titanium plates for the patients with MCSM, the surgical results were satisfying based on postoperative radiographical and functional evaluation and low complication rates. Statistical analysis showed that preoperative Nurick score was significantly related to surgical outcomes, which could be considered as an indicator for prognosis. The limitations of this study were its small patient sample size and a lack of comparator groups.
